# Our Invitation

**Published:** 1853-10

**Authors:** 


An Invitation.—Strenuous efforts are being made, by the Faculty and friends of the Medical College, in this city, to build up a Museum of Anatomical and Pathological specimens, connected with the Institution. We wish to form a valuable collection of specimens, which may not only be most useful, in practical illustrations to the course of Lectures before medical students, but may also be an object of profit and interest to the physician and naturalist, who may visit our city. The hundreds of physicians, who spend a few days in this city, in the course of every year, would be most agreeably and usefully entertained, by an annual visit to a well stored Museum. Already a noble beginning has been made—numerous valuable contributions have been received, both from resident physicians and those at a distance. We received a few days since, from the extremest part of the State, a valuable and very acceptable collection of preparations. We would hereby invite and urge our friends, and the friends of the Institution, wherever they may be, to favor the College, and aid the common cause of Medical education, by forwarding to us any such specimens of anatomical or pathological interest, as they may find it agreeable to furnish. All such specimens shall be most carefully preserved, with the name of the donor. We thus earnestly hope, by our own efforts, and the assistance of numerous friends, to lay the foundation of a collection which, increasing year by year, will eventually form a cabinet that shall be an honor to the Institution and the State.

